# Long-Term Safety of Anti-TNF Adalimumab in HBc Antibody-Positive Psoriatic Arthritis Patients: A Retrospective Case Series of 8 Patients

**DOI:** 10.1155/2013/410521

**Published:** 2013-03-27

**Authors:** R. Laurenti, F. Giovannangeli, E. Gubinelli, M. T. Viviano, A. Errico, L. Leoni, E. Ballanti, A. Migliore

**Affiliations:** ^1^Unità di Reumatologia, Istituto Dermopatico dell'Immacolata-IRCCS, Via Monti di Creta 104, 00165 Rome, Italy; ^2^Unità di Reumatologia, Ospedale S. Pietro Fatebenefratelli, Via Cassia 600, 00189 Rome, Italy; ^3^Unità di Dermatologia, Istituto Dermopatico dell'Immacolata-IRCCS, Via Monti di Creta 104, 00165 Rome, Italy; ^4^Unità di Gastroenterologia, Ospedale San Carlo, Via Aurelia 275, 00165 Rome, Italy

## Abstract

Immunosuppressive drugs commonly used in the treatment of psoriatic arthritis make patients more susceptible to viral, bacterial, and fungal infections because of their mechanism of action. They not only increase the risk of new infections but also act altering the natural course of preexisting infections. While numerous data regarding the reactivation of tuberculosis infection are available in the literature, poor information about the risk of reactivation or exacerbation of hepatitis viruses B and C infections during treatment with biologics has been reported. Furthermore, reported series with biological therapy included short periods of followup, and therefore, they are not adequate to verify the risk of reactivation in the long-term treatment. Our study evaluated patients with a history of hepatitis B and psoriatic arthritis treated with adalimumab and monitored up to six years. During the observation period, treatment was effective and well tolerated in all patients, and liver function tests and viral load levels remained unchanged.

## 1. Introduction

Immunosuppressive agents used in psoriatic arthritis (PsA) might have an effect on the natural course of coexisting infectious diseases or new developed infections. An association between the use of antitumor necrosis factor (TNF) and an increased risk of severe bacterial infections or reactivation of tuberculosis has been reported [1]. Long-term safety and efficacy of anti-TNF agents in patients with chronic hepatitis B or hepatitis C are not known [1]. There is increasing evidence demonstrating elevated serum levels of TNF-*α* in hepatitis-C-virus-(HCV-) infected patients compared with healthy controls, and a correlation exists between elevated TNF-*α* levels and serum alanine aminotransferase (ALT) levels. These findings suggest that TNF-*α* may be involved in the pathogenesis of hepatocyte destruction in chronic HCV infection. Case reports and a small prospective study indicate that anti-TNF therapy may be safe, and even beneficial, to use in chronic HCV [2–6]. In contrast to HCV, TNF-*α* may play a role in clearing and controlling hepatitis B virus (HBV). Elevated levels of TNF-*α* are observed in both the serum and hepatocytes of patients with chronic HBV, and TNF-*α* expression is markedly upregulated in acute-on-chronic liver failure in chronically HBV-infected patients [7, 8].

TNF-*α* is secreted by HBV-specific cytotoxic T lymphocytes (CTL) and seems to be synergizing with interferons in suppressing viral replication [9]. Animal studies show that TNF-*α* knockout mice have defects in the proliferative capacity of the HBV-specific CTL, suggesting that TNF-*α* may play a role in clearing or controlling HBV [10]. Therefore, inactivation of TNF-*α* could theoretically lead to enhanced viral replication and reactivate or worsen the disease [11].

The risk of HBV reactivation appears to be linked both to the phase of immunosuppression and to that of immune reconstitution. The risk of clinical events is mainly observed in active carriers of HBV but can also develop in the occult condition of infection which has been widely described in the literature in the last decade (Table 1) [12]. For patients with a known history of HBV who are HBsAg negative, the risk of reactivation appears to be significantly lower, but cannot be totally ruled out [13]. In the rheumatologic field, reports regarding the reactivation of HBV during the course of hydroxychloroquine, azathioprine, methotrexate, and other conventional DMARDs are episodic [14]. Safety and efficacy of anti-TNF agents in chronic hepatitis B are not known, and data available on reactivation of these viral infections are conflicting [15]. 

## 2. Patients and Methods

We enrolled eight patients, four females and four males, aged between 35 and 70 years, all suffering from PsA resistant and/or intolerant to conventional DMARDs, referred to our outpatients' clinics between 2006 and 2010. The average values of DAS28 and PASI before starting biologic agent were respectively, 6.49 ± 0.54 and 14 ± 15.62. None of the patients was aware of previous HBV infection nor of the period of contagion. All patients were HCV negative. 

Before starting immunosuppressive therapy, serological markers of HBV were evaluated in each patient. The cases in this study included 1 case of an inactive HBsAg carrier and 7 cases of HBcAb-positive cases, 6 of which can be considered as “past HBV infection.” All patients were negative for HBeAg. Patients' characteristics at baseline are shown in Table 2. 

All patients started the treatment with the anti-TNF adalimumab (Humira, Abbott Laboratories); only the inactive carrier was subjected to prophylactic therapy with lamivudine 100 mg/day [16], which was started 1 month before starting anti-TNF. Adalimumab was administered at the standard dose of 40 mg every 2 weeks.

## 3. Results

During the treatment period, we have carried out the evaluation of HBV markers and liver function initially after three months, and thereafter every six months; in all patients, throughout the treatment period, the HBV markers and liver function values remained stable. After the first six months each patient achieved clinical remission of both skin lesions and joint symptoms defined as DAS28 < 2.6 and 75% improvement in the PASI score.

The inactive carrier achieved clinical remission after the first few months of therapy with adalimumab; treatment was continued for 24 months and then stopped for the potential risk of reactivation of the viral disease and also because of the stable regression of psoriatic disease. Lamivudine was continued for further three months after the suspension of adalimumab.

## 4. Discussion

To date, only few studies regarding the use, efficacy, and tolerability of anti-TNF in psoriatic disease patients with previous HBV infection have been carried out and included a small number of patients (Table 3) [17–20].

In clinical practice, the guidelines for the use of biological drugs in PsA published in 2005 are usually considered [21]. According to these, HBV infection is considered a relative contraindication [21]. Therefore, the treatment is considered when absolutely necessary after evaluation of the risk of viral reactivation.

In fact, during the treatment with anti-TNF agents, several cases of transient viral reactivation, regressed with antiviral therapy, and rare cases of fulminant hepatitis have been reported, particularly in patients treated with infliximab [22]. 

It seems that the risk of viral reactivation during immunosuppressive therapy changes according to patients' serological category [23–27].

We can distinguish active carriers (HBsAg pos, HBV-DNA > 2000 IU/mL), inactive carriers (HBsAg pos, HBV-DNA < 2000 IU/mL), occult carriers (HBsAg neg, HBcAb pos), and patients with past HBV infection (HBsAg neg, HBcAb pos, and HBsAb pos).

The risk of reactivation appears to be greater in HBsAg-positive patients and lower, not exceeding 5%, in and HBsAg-negative HBcAb-positive patients (carriers or not of HBsAb) [19, 28].

Data in the literature are still conflicting. A report of 266 patients showed a higher incidence of abnormal liver function in 88 occult carriers treated with etanercept, infliximabs or adalimumab, compared with the control group [29].

Data regarding the appropriateness of biological treatments in patients with a history of hepatitis are contrasting, depending not only on the small number of patients treated but especially on the short period of treatment reported.

Our study represents a series of patients suffering from PsA with a history of hepatitis B treated with adalimumab up to six years. Since 2006, we followed 8 patients observing the serological pattern, liver function, and viral load throughout the treatment period.

Patients' serological pattern was not uniform: a patient was inactive carrier; other patients were occult carriers (among these 6 cases could be considered as past HBV infection). In all patients, the viral load was negative, and liver function was normal. In accordance with the guidelines of the European Association for the Study of Liver, HBsAg-positive patient was subjected to prophylaxis with lamivudine, which began before immunosuppression and continued for three months after therapy suspension [25].

Prophylactic treatment with lamivudine should start 2–4 weeks before the immunosuppressive therapy and continue for 6–12 months after the suspension of the same. In these patients, the assessment of transaminases and viral load is very important because the increase of the viral load over 10 times is associated to the possible development of resistance to lamivudine. Unlike cancer patients, who are treated with immunosuppressive drugs for short periods, rheumatic patients have to be treated for longer period, and this makes the role of antiviral prophylaxis still debated, as it is not clear what is the optimal period of treatment to avoid possible resistance induction. However, lamivudine seems to be effective in patients treated with anti-TNF, although the control groups are needed to confirm this finding. 

The 7 occult carriers enrolled had low viral load, and they did not receive any prophylactic drug as previously reported [11, 30]. To date, only the inactive carrier, which obtained stable clinical remission, discontinued treatment after 24 months, for the potential risk of viral disease reactivation. The other 7 patients are still in treatment and show no signs of viral reactivation or flare of joint or skin disease.

In our study, adalimumab administered for a long period has been proved effective and well tolerated in patients suffering from PsA. All the patients, at the beginning of anti-TNF treatment, exhibited normal liver function and a negative viral load (<200 IU/mL) due to a resolved viral disease. These conditions seem to be necessary to begin safe treatment with anti-TNF. However, other authors reported cases of reactivation in patients with the same characteristics.

To date, there is no certainty about the risk of reactivation, but, according to our findings, the relative safety of anti-TNF and the lack of direct hepatotoxicity encourage its use in PsA patients resistant or intolerant to conventional DMARDs and with occult or inactive viral disease. The limitations of our study concern the small number of patients which require studies on larger groups of patients. 

Our results, however, confirm the efficacy and safety of adalimumab, administered for long periods, in patients with PsA and past HBV infection.

## Figures and Tables

**Table 1:** Relevant virologic categories of hepatitis B infection.

Markers	Active carrier	Inactive carrier	Occult carrier
HBsAg	+	+	−
HBeAg	+/−	−	−
HBV-DNA	>2000 IU/mL	<2000 IU/mL	−
HBcAb IgM	+/−	−	−
HBcAb	+	+	+
HBeAb	+/−	+/−	+/−
HBsAb	−	−	+/−

**Table 2:** Patient's baseline characteristics.

Pt	Sex	Age	PsA duration (years)	DAS28	PASI	HBV DNA	HBsAg	HBsAb	HBcAb	HCV Ab	ALT	Drug	Class	Year of starting ADA
1	M	55	6	6.1	0	Neg	Neg	Pos	Pos	Neg	N	ADA	OC (PI)	2006
2	M	70	3	6.3	0	Neg	Pos	Neg	Pos	Neg	N	ADA	IC	2007
3	F	55	1	5.5	26.2	Neg	Neg	Pos	Pos	Neg	N	ADA	OC (PI)	2007
4	F	67	5	7.0	31.8	Neg	Neg	Neg	Pos	Neg	N	ADA	OC	2008
5	M	37	1	6.8	14.2	Neg	Neg	Pos	Pos	Neg	N	ADA	OC (PI)	2008
6	F	47	4	7.2	0	Neg	Neg	Pos	Pos	Neg	N	ADA	OC (PI)	2009
7	M	62	2	6.4	2.8	Neg	Neg	Pos	Pos	Neg	N	ADA	OC (PI)	2010
8	F	35	2	6.6	37.0	Neg	Neg	Pos	Pos	Neg	N	ADA	OC (PI)	2010

Pt: patient; Class: classification; ADA: adalimumab; N: normal; OC: occult carrier; PI: past infection; IC: inactive carrier.

**Table 3:** Previous reports about anti-TNF use in HBV active/inactive/occult carriers with psoriatic disease.

Study	Study type	PsA/Pso patients	Patients' categories	Premedication	Anti-TNF	Followup period	HBV reactivation
Cassano et al. 2011 [17]	R	34 PsA 28 PsO	OC	No	44 ETA 10 ADA 8 IFX	About 4 years	Reappearance of HBsAg without detectable HBV-DNA in 1 patient
Prignano et al. 2011 [18]	R	11 PsO	OC	No	ETA	7.8 months	No viral reactivation
Cho et al. 2012 [19]	R	5 PsA 2 PsO	2 IC 5 AC	1 patients had lamivudine and then entecavir	1 ADA 6 ETA	26.6 months (range 14–45)	3 patients had HBV reactivation
Fotiadou et al. 2011 [20]	R	7 PsO	IC	Lamivudine	3 ADA 3 ETA 1 IFX	6–24 months	1 patient receiving IFX had increase of viral load up to 600 IU/mL

Psa: psoriatic arthritis; PsO: psoriasis; R: retrospective; OC: occult carrier; IC: inactive carrier; AC: active carriers; ADA: adalimumab; ETA: etanercept; IFX: infliximab.
